# Minimal important change of the knee injury and osteoarthritis outcome score in patients with mild to moderate knee osteoarthritis – using three different anchor-based methods

**DOI:** 10.1016/j.ocarto.2025.100699

**Published:** 2025-10-29

**Authors:** Henriette Killingrød Lundquist, Britt Elin Øiestad, Joseph Sexton, May Arna Risberg, Nina Østerås

**Affiliations:** a Department of Physiotherapy, Oslo Metropolitan University, Norway; b Department of Rehabilitation Science and Health Technology, Oslo Metropolitan University, Norway; c Center for Treatment of Rheumatic and Musculoskeletal Diseases (REMEDY), Diakonhjemmet Hospital, Oslo, Norway; d Department of Sports Medicine, Norwegian School of Sport Sciences and Division of Orthopedic Surgery, Oslo University Hospital, Norway

**Keywords:** Osteoarthritis, Patient reported outcomes, Statistical methods

## Abstract

**Objective:**

To estimate Minimal Important Change (MIC) for improvement in the Knee injury and Osteoarthritis Outcome Score (KOOS) in patients with mild to moderate knee osteoarthritis (OA), using three recommended anchor-based methods, and examine how methodological choices influence these estimates.

**Design:**

Secondary analysis of a three-arm randomized controlled trial. KOOS and a Global Rating of Change (GROC) scale were collected at baseline and 4-month follow-up. MIC values were estimated using predictive modeling, Mean Change, and Receiver Operating Characteristic (ROC) methods. Sensitivity analyses assessed the impact of different anchor cut-offs.

**Results:**

Data were available for 131 patients undergoing non-surgical treatment (mean age 57.4 years, 50 ​% female). At follow-up, 19 ​% reported important improvement. Using the predictive modeling method, which allows adjustment for the low proportion of patients reporting important improvement, MICs were 11.3 (Pain), 12.1(Symptoms), 10.2 (ADL), 15.5 (Sport/Rec) and 13.2 (QoL). The Mean Change method yielded comparable MICs (range 10.6–16.1), but due to its reliance on a small subgroup, it is generally considered less robust and showed wider CIs in our sample. ROC-based MICs ranged from −0.4 to 12.5 and were associated with wide CIs, and high misclassification rates. Sensitivity analyses showed lower MICs with broader improvement definitions were used.

**Conclusion:**

MIC estimates for KOOS varied considerably by method. Predictive modeling yielded the most precise MIC estimates and should be considered for future research, particularly when the proportion of improved patients deviates from 50 ​%. These results also highlight the importance of methodological transparency for interpreting PROMs in non-surgical knee OA treatment.

## 1 Introduction

Patient-reported outcome measures seek to capture symptoms, functional status, and health-related quality of life (QoL) directly from the patient's perspective [1].The Knee injury and Osteoarthritis Outcome Score (KOOS) is a widely used PROM for patients with knee osteoarthritis (OA). Developed in the 1990s, KOOS assesses pain, knee symptoms, function and knee-related QoL [2]. It has been shown to be reliable and valid for both younger and older adults with knee injuries and/or OA [3,4].

A key challenge when interpreting change in KOOS scores, whether in clinical practice or randomized controlled trials (RCT), is determining whether a statistically significant change also reflects an important change for patients. To interpret the impact of change scores, calculation of a Minimal Important Change (MIC) is helpful. MIC is defined as the smallest change in PROM score that patients perceive as an important change [5].

While several studies have reported MIC values for KOOS, most have studied populations undergoing surgical interventions [[6], [7], [8]]. Only a limited number of studies have investigated MIC in non-surgical interventions for knee OA, and these often rely on single methods and/or other shorter versions of the KOOS [[9], [10], [11], [12]].

MIC values are found to vary by population and baseline severity [13], and are dependent on the methodological approach [14]. Understanding how different methodological and statistical approaches contribute to the variability in MICs is particularly relevant for non-surgical management of knee OA, where treatment effects are often more subtle and interpretation of Patient-reported outcome measures is challenging. To our knowledge, no prior study has systematically compared multiple anchor-based methods for estimating MIC in patients with *mild to moderate knee OA* receiving non-surgical management. This represents an important gap, as non-surgical treatment is widely recommended as first-line management, and reliable MIC values are needed to interpret clinical outcomes and evaluate interventions in this context.

Therefore, the aim of this study was to estimate MIC values for KOOS in patients with mild to moderate knee OA using three recommended anchor-based methods (predictive modeling, mean change, and ROC) and to examine how methodological choices, such as anchor cut-off definitions, influence these estimates.

## 2 Method

This study used data from baseline and the 4-month follow-up (post-intervention) of a three-arm RCT with two exercise interventions aiming to improve QoL and knee function [15,16] (Clinical trials NCT01682980). For the present analyses, data from all three trial arms (strength exercise, aerobic exercise, and usual care) were pooled and analyzed together. This approach was chosen because the aim of the current study was to estimate MIC values across a broader spectrum of patients with mild to moderate knee OA, rather than to evaluate the efficacy of a specific intervention. Pooling the groups allowed us to capture values for a mix of interventions, including strength training, cycling and usual care enhancing the generalizability of our findings beyond a single intervention context.

Reporting of this secondary analysis of RCT data follows the STrengthening the Reporting of Observational studies in Epidemiology guidelines [17]. The Regional Ethical Committee in the Health Region South-East in Norway approved the study (REK 2012/334), and all patients signed informed consent prior to the baseline assessment.

### 2.1 Participants

The patients were recruited from primary health care, orthopedic departments at three hospitals in the greater Oslo area, and from newspaper advertisement from April 2013 to March 2020. The patients were randomly allocated to strength exercise (n ​= ​55), aerobic exercise (n ​= ​56) or usual care (n ​= ​57). Both exercise groups started with a two-week preparation phase to adapt to the exercise program and were then told to exercise 2–3 times per week for 12 weeks. Participant flow chart and further details of the interventions are previously presented in detail [15].

### 2.2 Collected data

Data for patient demographics (e.g., age, gender, height, weight, years diagnosed with osteoarthritis, educational level, frequency of physical activity, known heart disease, and knee pain last week (0–10)) were self-reported at the time of recruitment and before random group allocation (baseline). Height and weight were also objectively measured at baseline [15]. Body Mass Index was calculated with the formula; weight (kg)/height (meters)^2^ [18]. All patients were asked to complete questionnaires at baseline and at the 4-month follow-up, in addition an anchor question at follow-up. No interim assessments were performed between the baseline measurement and the 4-month follow-up.

### 2.3 Inclusion and exclusion criteria

The patient inclusion criteria were age 35–70 years with confirmed radiographic knee OA, Kellgren and Lawrence grade 2 and 3 (mild to moderate) [19,20], symptomatic knee OA where patients had to fulfill 3/4 of the American College of Rheumatology clinical criteria (stiffness <30 ​min, crepitus, osteophytes, pain the last day of the last month) [21], and having no other serious mental or physical illnesses preventing them from participating in the RCT [15]. Patients were excluded if they self-reported BMI>35 ​kg/m^2^ at the time of recruitment, were scheduled for surgery within 6 months, already participated in structured weekly training, had known serious musculoskeletal impairments in the lower extremities or low back, had serious coronary diseases or cancer, or had prostheses in the lower extremities, or did not speak Norwegian [15]. In the present study, patients were not included in the analyses if they had missing data on KOOS or anchor question at follow-up.

### 2.4 The questionnaire and anchor-based question

Patients completed the Norwegian version of KOOS at baseline and the 4-month follow-up. KOOS includes five subscales containing 4–17 items measuring pain, other symptoms, Activities of Daily Living (ADL), function in sports and recreation and knee related QoL. All items were scored 0–4. The scores were then normalized to a score from 0 to 100 for each subscale independently, 0 indicating extreme symptoms and 100 representing no symptoms [2]. Missing items in the KOOS subscales were handled following the guidelines, allowing calculation of subscale scores when over 50% of the items were completed [22].

At the 4-month follow-up, a Global Rating of Change (GROC) was completed, asking “H*ow are your knee complaints now compared to the previous assessment?”.* The 7- point Likert scale had the response categories: 1 ​= ​completely recovered, 2 ​= ​much better, 3 ​= ​a little bit better, 4 ​= ​no change, 5 ​= ​a little bit worse, 6 ​= ​much worse, and 7 ​= ​worse than ever.

### 2.5 Statistical methods

Statistical analyses were performed using Statistical Package for Social Sciences (IBM© SPSS© Statistics version 22) and STATA software 17. Due to the nature of the research question, both exercise groups and the usual care group were included in the dataset. Descriptive characteristics of the patients were presented as absolute number and percentages for categorical variables, and as mean and standard deviation or median and interquartile range for continuous variables. KOOS change scores were calculated by subtracting baseline score from their respective subscale at the 4-month follow-up. Mean change scores were presented for each GROC category, and the distribution was illustrated with boxplots.

To evaluate the anchors' validity, Spearman's correlations were used to investigate the correlation between the KOOS change scores and each of the 1–7 scales of the anchor (GROC). Due to inconsistency in the literature, no predefined correlation threshold was set prior to the analysis [23,24].

### 2.6 Anchor-based MIC methods

MIC values for improvement were estimated using three anchor-based methods, in line with recommendations from Outcome Measures in Rheumatology (OMERACT) [25]. In anchor-based approaches, changes in PROM scores are linked to an external anchor that captures patients’ perceived importance of change [5]. We used a global GROC as the anchor in all three methods. The GROC response categories were used to dichotomize the patients into an importantly improved group and a not importantly improved group. There is no consensus on the optimal GROC cut-off [26]. In this study, patients were classified as importantly improved when responding “completely recovered” or “much better”, and as “not importantly improved” if they selected any other response. We chose this stricter cut-off to ensure that the importantly improved group reflected meaningful improvement, as responses such as *“a little better”* may capture minor changes not considered clinically relevant in patients with knee OA. Because the purpose of this study was to estimate MIC for improvement, no separate group for importantly deteriorated was defined, and patients reporting deterioration were included in the “not-importantly improved” group.

The simplest and most prevalent anchor-based method is the Mean Change method (MIC_meanchange_). In this approach, the MIC is defined as the mean change in score on the measurement instrument in the subgroup of patients who are *minimally importantly changed*, according to the anchor [5]. In our study, MIC values with 95 ​% confidence intervals were calculated from the KOOS mean change score within the subgroup of patients responding, “much better” (minimal important improvement) to the anchor question. As previously noted in the literature, this method has important drawbacks, including reliance on small subgroups and the risk that the mean change does not truly reflect a minimal important threshold [27]. If the anchor is not fully accurate, MIC_meanchange_ estimates are also more prone to bias compared with other anchor-based methods [27].

The Receiver Operating Characteristic (ROC) is based on the ability of the anchor to discriminate between importantly changed and not importantly changed patients [14]. The Youden criteria was used to identify the optimal MIC values in the ROC analysis (MIC_roc_), for which the sum of sensitivity and specificity reached its maximum.

The predictive modeling method (MIC_pred_) was introduced in 2015 showing more precise values than the ROC analysis [14]. The MIC_pred_ method uses logistic regression to estimate the MIC value, with the anchor (“importantly improved” or “not importantly improved”) as the binary dependent variable and the PROM change score as the independent variable. MIC_pred_ is then set to the PROM change score which is estimated as equally likely in the improved and not-improved groups (i.e the change score corresponding a likelihood ratio of 1) [14]. At the KOOS change score corresponding to a LR+ ​= ​1 (the MIC_pred_ estimate), the chance of a patient considering themselves “importantly improved” equals that of a randomly selected patient. However, for any change score exceeding this value, there is a greater than random chance that the patient would consider their condition as “importantly improved.” This is why the MIC_pred_ is regarded as a lower bound for important change, i.e. a MIC. Both MIC_pred_ and MIC_roc_ can be biased if the proportion of improved patients differs from 50 ​% [28]. The predictive method enables adjusting for the proportion of improved patients using the following equation: MIC_adjusted_ ​= ​MIC_pred_ – (0.090 ​+ ​0.103 ∗ Cor) ∗ SD_change_ ∗ log-odds(imp) [28]. Cor represents the point biserial correlation between the PROM change score and the anchor. SD_change_ represents the standard deviation of the change score, and log-odds(imp) represents the natural logarithm of (proportion improved/(1-proportion improved)) [28]. Further, the predictive MIC allows for testing and estimation of modifying factors such as baseline severity [14].

Bootstrapping with 1000 random replications was used to determine 95 ​% CIs for the MIC_roc_ and the MIC_pred_.

### 2.7 Sensitivity analyses

To investigate if the GROC cut-off value and following categorization of being importantly improved had a major impact on the results, analyses were performed with a different cut-off, by including patients reporting ‘a little bit better’ together with the patients reporting ‘much better’ or ‘completely recovered’ in the importantly improved group.

Further, to be able to investigate the impact of very high or very low symptoms or function scores at baseline, formulas were made for calculating MIC_pred_ adjusted for KOOS baseline values for the two different definitions of importantly improved.

## 3 Results

**Fig. 1:**
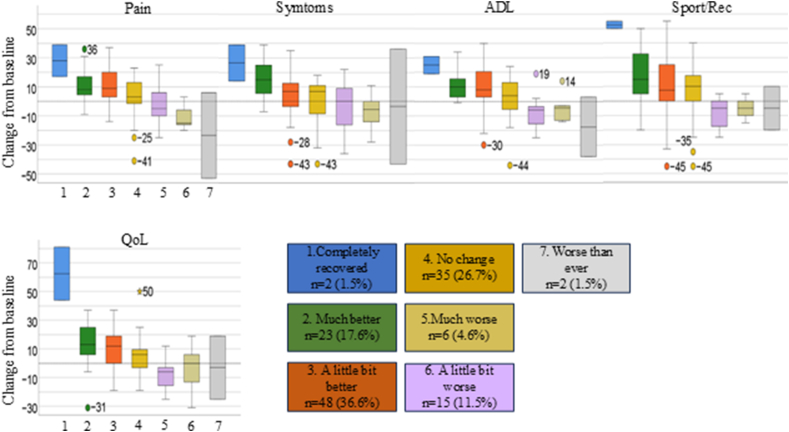
KOOS mean change scores by anchor question response category ranging from completely recovered to worse than ever. Horizontal bars represent the median, the box represents the IQR, and the whiskers represent the highest and lowest scores. Circles indicate mild outliers that are more than 1.5 x IQR values and asterisks represent extreme outliers that are more than 3.0 x IQR values. The numbers by the circles and asterisks represent their actual change scores.

The trial randomized 168 patients. Of those, 131 (78.0 ​%) patients completed KOOS and the anchor question at the 4-month follow-up and were included in the present analyses. Included patients were on average 57.4 years of age, 49.6 ​% were female, and mean Body Mass Index was 28.9 (SD 4.3) kg/m^2^ (Table 1). Baseline characteristics for included versus non-included patients are presented in Supplementary Table S1. These data show no meaningful differences between the two groups, supporting the generalizability of the sample analyzed. At the 4-month follow-up, 19 ​% (n ​= ​25) evaluated themselves as importantly improved. The mean change scores were higher for patients reporting an improvement and negative for those responding ‘a little bit worse’ or worse on the GROC (Table 2). In general, there was considerable overlap between the distribution of the individual change scores for each category of the GROC (Fig. 1).

**Table 1:** Baseline characteristics of the included patients (n ​= ​131).

Female subjects, n (%)	65 (49.6)
Age (year), mean (SD)	57.4 (6.8)
Years diagnosed with OA, median (IQR)	2.0 (4)^a^
Body Mass index (kg/m^2^), mean (SD)	28.9 (4.3)
Education, n (%)	
Less than high school	15 (11.5)
High school	42 (32.1)
College/University	74 (56.5)
Physical activity frequency, n (%)	
≤ ​Once a week	54 (41.2)
> ​2–3 times per week	77 (58.8)
Self-reported known heart disease, n (%)	36 (27.5)^b^
Knee pain last week (0–10), mean (SD)	4.9 (2.1)
KOOS scores, mean (SD)	
Pain	53.9 (18.2)
Symptoms	58.3 (18.4)
ADL	62.9 (20.6)
Sport/Rec	27.5. (21.3)
QOL	32.5 (17.5)

a 7 missing data.

b 1 missing data; SD, Standard Deviation; OA, Osteoarthritis; IQR, Interquartile Range; KOOS, Knee injury and Osteoarthritis Outcome Score; ADL, Activities of Daily Living; Sport/Rec, Sport and Recreational Function; QOL, Quality of Life.

**Table 2:** Mean change scores for KOOS subscales in the different response categories on the Global Rating of Change (GROC).

GROC	n (%)	Pain mean change score (SD)	Symptoms mean change score (SD)	ADL mean change score (SD)	Sport/Rec mean change score (SD)	QoL mean change score (SD)
Total sample	131 (100)	6.0 (15.0)	3.9 (17.4)	5.1 (15.5)	7.8 (20.0)	7.0 (16.1)
Completely recovered	2 (1.5)	28.0 (15.6)	26.5 (17.7)	25.0 (8.5)	52.5 (3.5)	62.5 (26.2)
Much better	23 (17.6)	11.6 (11.3)	14.9 (14.1)	10.6 (10.1)	16.1 (18.5)	13.5(16.1)
A little bit better	48 (36.6)	10.9 (12.8)	4.2 (16.7)	10.0 (16.3)	10.5 (20.8)	9.7 (11.1)
No change	35 (26.7)	2.5 (13.6)	0.5 (14.8)	2.5 (13.5)	5.9 (17.9)	4.8 (13.0)
A little bit worse	15 (11.5)	−1.9 (13.0)	−3.7 (17.1)	−8.5 (11.1)	−8.0 (10.5)	−8.7 (9.6)
Much worse	6 (4.6)	−11.5 (8.5)	−7.0 (13.6)	−4.2 (10.1)	−5.0 (7.7)	−3.2 (17.1)
Worse than ever	2 (1.5)	−23.5(41.7)	−3.5 (55.9)	−17.5(29,0)	−5.0 (21.2)	−3.0 (31.1)

KOOS, Knee injury and Osteoarthritis Outcome Score; SD, Standard Deviation; ADL, Activities of Daily Living; Sport/Rec, Sport and Recreational Function; QoL, Quality of Life.

### 3.1 MIC improvement values

The correlations between KOOS change scores and the anchor question were 0.43 for Pain, 0.35 for Symptoms, 0.44 for ADL, 0.41 for Sport/Rec and 0.46 for QoL.

**Fig. 2:**
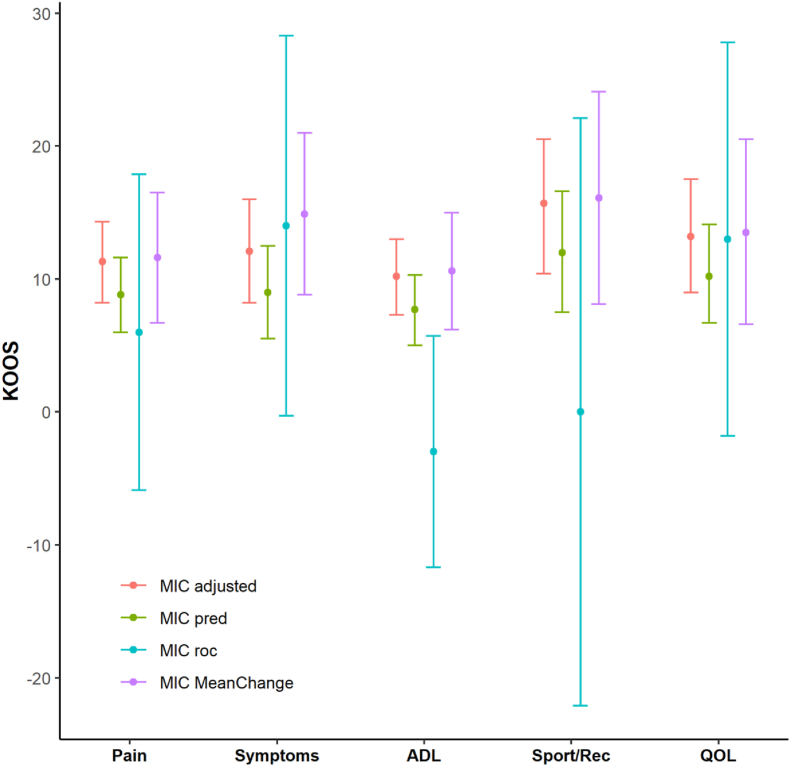
MIC values for KOOS subscales at 4 months follow-up obtained by the different anchor-based methods. Dots represent the MIC estimates, and the whiskers represent the 95 ​% CI.

**Fig. 3:**
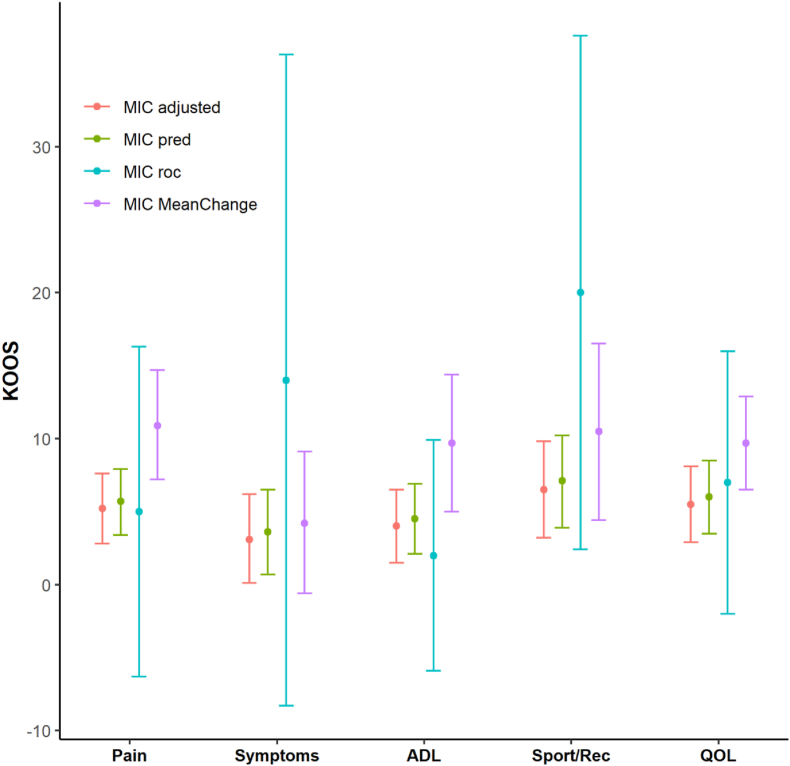
MIC values for KOOS subscales at 4 months follow-up with a cut-off including the response category ‘a little bit better’ in the importantly improved group. Dots represent the MIC estimates, and the whiskers represent the 95 ​% CI.

**Fig. S1:**
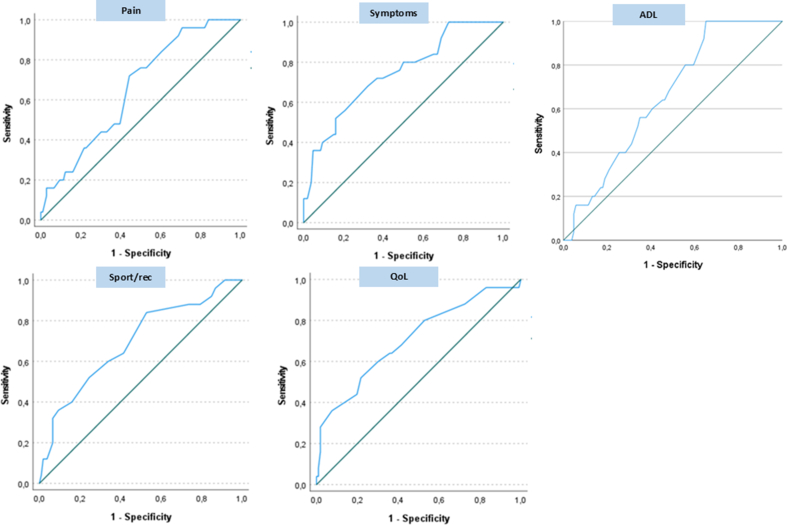
Receiver Operating characteristic curve for each KOOS subscales with the total sample of participants, Area under the curves ranging from 0.65 to 0.75. Abbreviations: ADL, Activities of Daily Living; Sport/rec, Sport and Recreational Function; QOL, Quality of Life.

The MIC values varied between the different anchor-based methods (Fig. 2). MIC_pred_ improvement values, adjusted for the small proportion of improved patients, were higher than the unadjusted MIC values. The MIC values determined by the Mean Change method were comparable to those from MIC_adjusted_ but had wider 95 ​% CIs. The ROC method yielded generally flattened ROC curves and low areas under the curve (AUC). Only the subscales Symptoms and QoL reached an AUC >0.7 (see Supplementary Fig. S1). This resulted in a wide range of cut-off points associated with approximately the same degree of misclassification. In addition to the visual presentation in Fig. 2, Table 3 summarizes MIC values with 95 ​% CIs for all three methods to further enhance clarity and comparability. Sensitivity analysis using a broader cut-off for “importantly improved” resulted in lower MIC values for all methods, except for the ROC method (Fig. 3 and Table 4).

**Table 3:** MIC values for KOOS at 4 months follow-up obtained by the different anchor-based methods.

KOOS	MIC_adjusted_^a^ (95 ​% CI)^b^	MIC_pred_^c^ (95 ​% CI)^b^	MIC_roc_^d^ (95 ​% CI)^b^	MIC_MeanChange_^e^ (95 ​% CI)
Pain	11.3 (8.2; 14.3)	8.8 (6.0; 11.6)	6 (−5.9; 17.9)	11.6 (6.7; 16.5)
Symptoms	12.1 (8.2; 16.0)	9.0 (5.5; 12.5)	14 (−0.3; 28.3)	14.9 (8.8; 21.0)
ADL	10.2 (7.3; 13.0)	7.7 (5.0; 10.3)	−3 (−11.7; 5.7)	10.6 (6.2; 15.0)
Sport/Rec	15.7 (10.4; 20.5)	12.0 (7.5; 16.6)	0 (−22.1; 22.1)	16.1 (8.1; 24.1)
QOL	13.2 (9.0; 17.5)	10.2 (6.7; 14.1)	13 (−1.8, 27.8)	13.5 (6.6; 20.5)

a Estimates obtained from the predictive modeling method adjusted for proportion of improved.

b 95 ​% confidence intervals calculated with 1000 bootstrap replications.

c Estimates obtained from unadjusted predictive modeling method.

d Estimates obtained from Receiver Operating Characteristic (ROC) method.

e Estimates obtained from Mean Change method; MIC, Minimal Important Change; KOOS, Knee injury and Osteoarthritis Outcome Score; ADL, Activities of Daily Living; Sport/Rec, Sport and Recreational Function; QOL, Quality Of Life.

**Table 4:** MIC values for KOOS at 4 months follow-up with a cut-off including the response category ‘a little bit better’ in the importantly improved group.

KOOS	MIC_adjusted_^a^ (95 ​% CI)^b^	MIC_pred_^c^ (95 ​% CI)^b^	MIC_roc_^d^ (95 ​% CI)^b^	MIC_MeanChange_^e^ (95 ​% CI)
Pain	5.2 (2.8; 7.6)	5.7 (3.4; 7.9)	5 (−6.3; 16.3)	10.9 (7.2, 14.7)
Symptoms	3.1 (0.1; 6.2)	3.6 (0.7; 6.5)	14 (−8.3; 36.3)	4.2 (−0.6; 9.1)
ADL	4.0 (1.5; 6.5)	4.5 (2.1; 6.9)	2 (−5.9; 9.9)	9.7 (5.0; 14.4)
Sport/Rec	6.5 (3.2; 9.8)	7.1 (3.9; 10.2)	20 (2.4; 37.6)	10.5 (4.4; 16.5)
QOL	5.5 (2.9; 8.1)	6.0 (3.5; 8.5)	7 (−2.0, 16.0)	9.7 (6.5; 12.9)

a Estimates obtained from the predictive modeling method adjusted for proportion of improved.

b 95 ​% confidence intervals calculated with 1000 bootstrap replications.

c Estimates obtained from unadjusted predictive modeling method.

d Estimates obtained from Receiver Operating Characteristic method.

e Estimates obtained from Mean Change method; MIC, Minimal Important Change; KOOS, Knee injury and Osteoarthritis Outcome Score; ADL, Activities of daily living; Sport/Rec, Sport and Recreational Function; QOL, Quality Of Life.

## 4 Discussion

In the present study, 19 ​% of the patients with knee OA perceived their knee complaints to be importantly improved at the 4-month follow-up. MIC values varied considerably across the three anchor-based methods. The predictive modeling method appeared most appropriate, producing MIC estimates with narrower 95 ​% confidence intervals than the Mean Change and ROC methods, in line with previous research [7,8]. Importantly, this method also allows adjustment for the low proportion of improved patients, resulting in higher MIC values as expected when improvement rates deviate from 50 ​% [28].

The ROC method produced less interpretable results. For example, the Activities of Daily Living (ADL) subscale yielded a negative MIC, and several subscales had CIs crossing zero, and it does not make sense that a MIC can be both positive and negative [29]. AUC values were generally low (<0.7 for most subscales), indicating poor discrimination between importantly improved and not improved patients. Such instability is consistent with previous studies showing that the ROC-based MIC estimates are biased when the proportion of improved patients deviate from 50 ​% [7,14].This bias cannot be corrected within the ROC framework, whereas the predictive modeling method can adjust for it and generally yields more precise estimates [28] Given these limitations, MIC_ROC_ values are not recommended for further use in this context.

The Mean Change method yielded MIC values close to those from the MIC_adjusted_ in the main analysis, but with wider CIs. This similarity likely reflects that the subgroup reporting “much better” comprised most of those classified as importantly improved. Sensitivity analyses using a broader cut-off (including “a little better”) resulted in larger MIC_MeanChange_ than MIC_adjusted_ for all subscales. This is to be expected, as MIC_pred_ values are determined using the whole sample, whereas MIC_MeanChange_ reflects the average change within a subgroup in which all patients reported minimal important improvement on the GROC [27]. Furthermore, this method is more vulnerable to bias from anchor inaccuracy and less suitable for defining responders compared with the more robust MIC_pred_ [7,27].

Because MIC values varies with different methodological approaches [14], we compared our findings to other studies using anchor-based approaches. A recent systematic review identified two studies that determined MIC values for KOOS after non-surgical interventions [30]. One reported MICs for the total KOOS [9], and another for the shorter KOOS-PS and KOOS-QoL [10]. Median MICs for KOOS pain were 12.4, ADL 8.4, and QoL 9.8, with ADL and QoL being lower than our results. Mills et al. [9] applied a minimum correlation threshold and only reported MICs for certain subscales, which along with differences in interventions, follow-up time, and the use of ROC and/or Mean Change method may explain discrepancies.

Only one prior study has used predictive modeling for MIC values in knee OA after participating in digital first-line treatment, including education and exercise [12], reporting much lower MICs (1.5–3.6 points). That study used KOOS-12, which excludes challenging Sport and Recreation items, such as running, jumping, and kneeling. These activities may be difficult to improve in older patients with knee OA and could explain why the present study estimated higher MIC values. Two further studies have used predictive modeling for total KOOS, but the patients had more acute conditions [7,8].

There is no consensus on the optimal anchor cut-off for defining important improvement [26,30]. Our stricter cut-off likely contributed to the low proportion of improved patients and should be considered when comparing with studies using broader cut-off definitions. Mills et al. [9], for example, included category 3 “slightly improved”, while Cronström et al. [12] used anchors that explicitly specified importance of change. Notably, this category corresponded to option 2/7, meaning that, as in our study, only responses in options 1 and 2 were classified as importantly improved. Although we adopted a stricter cut-off to ensure that the MIC reflected clinically important change, it could be argued that, for patients with knee OA and chronic symptoms, even “a little better” might be meaningful. This highlights how cut-off choices influence MIC estimates.

The anchor in this study was global rather than domain-specific, which may reduce validity compared to domain-specific anchors [31]. Correlations between anchor responses and KOOS change scores were moderate (0.35–0.46), exceeding the >0.30–0.35 threshold suggested by Revicki et al. [24] but below the >0.50 recommended by de Vet et al. [23], and comparable to studies applying predictive modeling with domain-specific anchors [7,12]. Moderate correlations may partly reflect recall bias or response shift, where patients adapt and reassess their health over time [32,33]. GROC ratings often correlate more strongly with post-scores than change scores, suggesting current health status influences responses [34]. However, this was not investigated in this study.

The MIC estimates should also be interpreted in relation to the minimal detectable change of the PROM [35,36]. Our predictive MIC values did not exceed reported minimal detectable change values for KOOS in knee OA [3,37], suggesting some changes may not be distinguishable from measurement error. Differences in patient populations, such as baseline severity and treatment context, likely contribute to these discrepancies. Following the reasoning of de Vet & Terwee [38], the proposed MDCs for KOOS may fail to detect minimally important changes in this sample.

### 4.1 Strengths and limitations

A major strength of this study is the use of recommended anchor-based methods [25], and the direct comparison of three approaches, including the predictive modeling method. This yields more accurate estimates, particularly when the proportion of improved patients deviates from 50 ​% [14,27].

A key limitation of this study is the relatively low proportion of patients reporting important improvement (19 ​%), since this has been found to increase the risk of bias compared to equal-sized groups of improved and not improved [28]. According to Terwee et al. [27], the sample size of an MIC study should be at least 100 patients, and ideally about 50 ​% should be in the improved group. The percentage of improved patients should be reported, and if this percentage deviates from 50 ​%, the adjusted MIC_predict_ should be used. We followed this recommendation, but the low proportion may still reduce the stability of our MIC estimates. Therefore, throughout our results and conclusions, we emphasize and rely on the values derived from the adjusted MIC_predict_.

As already discussed, several factors may explain the relatively low proportion of patients reporting important improvement. One additional factor is the decision to include both the usual care group and those who worsened in the analysis. Although our aim was not to evaluate intervention efficacy, but rather to estimate MIC values across a broader spectrum of patients with mild to moderate knee OA, including these groups may have influenced perceived improvement. We did not perform separate ROC analyses excluding patients who worsened, though this is sometimes recommended [7]. For predictive modeling, we followed Terwee et al. [27] and included the full sample.

We did not estimate MICs for deterioration, despite 18 ​% reported worse outcomes (categories 5–7 on the GROC scale). Furthermore, we did not define a cut-off for important deterioration, and these patients were included in the "not importantly improved" group in all analyses. Our sample also included a higher proportion of male subjects than other non-operative knee OA studies, and compared to the world prevalence, which may affect generalizability. Finally, we did not analyze baseline severity, even though it is known to influence MICs. To mitigate this, we provided formulas enabling MIC calculation when baseline score is known (Table 5).

**Table 5:** Formulas for calculating MIC_adjusted_ for individual KOOS baseline values with different definitions of ‘importantly improved’.

KOOS subscale	MIC_adjusted_ for importantly improved^a^	MIC_adjusted_ for importantly improved^b^
Pain	24.8–0.3∗KOOS pain baseline	16.2–0.2∗KOOS pain baseline
Symptoms	31.4–0.4∗KOOS symptoms baseline	25.3–0.4∗KOOS symptoms baseline1
ADL	33.5–0.4∗KOOS ADL baseline	15.6–0.2∗KOOS ADL baseline
Sport/Rec	22.3–0.3∗KOOS Sport/Rec baseline	14.1–0.3∗ KOOS Sport/Rec baseline
QoL	20.8–0.3∗KOOS QoL baseline	12.0–0.2∗ KOOS QoL baseline

a Importantly improved defined as those responding, “much better” and “completely recovered” on the Global Rating of Change (GROC).

b Importantly improved defined as those responding, “a little bit better”, “much better” and “completely recovered” on the GROC.

In summary, MIC estimates for KOOS improvement in patients with mild to moderate knee OA varied considerably depending on the method used. Predictive modeling, particularly when adjusted for the low proportion of improved patients, yielded the most precise and robust estimates. These findings provide reference points for future research on non-surgical interventions in knee OA and highlight the importance of methodological transparency when interpreting PROM change scores. MIC values should be interpreted with awareness of both methodological choices and the specific sample characteristics studied.

## Authors contributions

Henriette Killingrød Lundquist: Writing - original draft, Visualization, Writing - review & editing, Formal analysis, Methodology, Conceptualization.

Britt Elin Øiestad: Principal Investigator, Writing - review & editing, Conceptualization, Data curation, Supervision, Funding acquisition, Investigation, Resources, Methodology.

Joseph Sexton: Methodology, Writing - review & editing, Formal analysis.

May Arna Risberg: Conceptualization, Writing - review & editing, Methodology, Investigation.

Nina Østerås: Supervision, Writing - review & editing, Methodology, Conceptualization, Formal analysis.

## Declaration of Generative AI in scientific writing

Nothing to disclose.

## Role of the funding source

The research Council of Norway funded the first 3-year of the randomized controlled trial (Ref. H10/213335). Oslo Metropolitan University and Oslo University Hospital funded the project through research positions to the PI and research coordinator. We have not received any financial support or benefits from commercial sources.

## Declaration of competing interests

All authors declare no possible conflict of interest related to the work or submission.

## Footnotes

Appendix A Supplementary data to this article can be found online at https://doi.org/10.1016/j.ocarto.2025.100699.
