# Supplementary material for: Minimal important change of the knee injury and osteoarthritis outcome score in patients with mild to moderate knee osteoarthritis – using three different anchor-based methods
Source: Osteoarthr Cartil Open. 2025 Oct 29;7(4):100699. doi: 10.1016/j.ocarto.2025.100699 (PMC12685517; doi:10.1016/j.ocarto.2025.100699)
Supplement: Multimedia component 1 [file mmc1.docx]

| **Supplementary Table S1:** Baseline characteristics of the included participants (n=131) and the excluded non-responders on the Global Rating of Change (GROC) (n=37) | | | | | |
| --- | --- | --- | --- | --- | --- |
|  | Included  n=131 | Excluded n=37 | Mean difference (95%CI) | p-value | |
| Female, n (%) | 65 (49.6) | 20 (54.1) |  | 0.69 | |
| Age (year), mean (SD) | 57.4 (6.8) | 56.5 (8.5) | 0.9 (3.5,1.8) | 0.50 | |
| Years diagnosed with OA, median (IQR) | 2.0 (4) ^a^ | 2.0 (6) ^b^ |  | 0.70 | |
| Body Mass Index (kg/m^2^), mean (SD) | 28.9 (4.3) ^c^ | 28.8 (4.1) ^c^ | 0.1 (-1.7,1.5) ^c^ | 0.88 ^c^ | |
| Education, n (%) | |  |  | 0.76* | |
| Less than high school ^c^ | 15 (11.5) ^c^ | 3 (8.1) ^c^ |  |  | |
| High school ^c^ | 42 (32.1) ^c^ | 14 (37.8) ^c^ |  |  | |
| College/University ^c^ | 74 (56.5) ^c^ | 19 (51.4) ^c^ |  |  | |
| Physical activity frequency, n (%)^c^ | |  |  | 0.27 ^c^ | |
| ≤ once a week | 54 (41.2) | 19 (51.4) |  |  | |
| > 2-3 times per week | 77 (58.8) | 18 (48.6) |  |  | |
| Self-reported known heart disease, n (%) | 36 (27.5) | 8 (21.6) |  | 0.46 |  |
| Knee pain last week (0-10), ^c^ mean (SD) | 4.9 (2.1) ^c^ | 5.1 (2.1) ^b^ | 0.13 (-0.7,0.9) ^c^ | 0.76 ^c^ | |
| KOOS scores, mean (SD) | |  |  |  | |
| Pain | 53.9 (18.2) | 55.1 (19.9) | 1.2 (-5.7,8.0) | 0.74 | |
| Symptoms | 58.3 (18.4) | 61.2 (18.4) | 2.9 (-3.8,9.7) | 0.39 | |
| ADL | 62.9 (20.6) | 64.2 (21.9) | 1.2 (-6.4,8.9) | 0.75 | |
| Sport/Rec | 27.5 (21.3) | 28.3 (23.6) | 0.8 (-7.3,8.8) | 0.85 | |
| QOL | 32.5 (17.5) | 35.4 (20.9) | 0.2 (-6.6,6.9) | 0.96 | |
| ^a^ 7 missing data; ^b^ 2 missing data; ^c^ 1 missing data; SD, Standard Deviation; CI; Confidence Interval; OA, Osteoarthritis; IQR, Interquartile Range; KOOS, Knee injury and Osteoarthritis Outcome Score; ADL, Activities of Daily Living; Sport/Rec, Sport and Recreational Function; QOL, Quality of Life; *1 cell has expected count less than 5. | | | | | |
